# Concurrent Infection with Clade 2.3.4.4b Highly Pathogenic Avian Influenza H5N6 and H5N1 Viruses, South Korea, 2023

**DOI:** 10.3201/eid3006.240194

**Published:** 2024-06

**Authors:** Gyeong-Beom Heo, Yong-Myung Kang, Se-Hee An, Yeongbu Kim, Ra Mi Cha, Yunyueng Jang, Eun-Kyoung Lee, Youn-Jeong Lee, Kwang-Nyeong Lee

**Affiliations:** Seoul National University, Seoul, South Korea (G.-B. Heo);; Animal and Plant Quarantine Agency, Gimcheon, South Korea (G.-B. Heo, Y.-M. Kang, S.-H. An, Y. Kim, R.M. Cha, Y. Jang, E.-K. Lee, Y.-J. Lee, K.-N. Lee)

**Keywords:** influenza, highly pathogenic avian influenza virus, viruses, poultry, duck, H5N6, H5N1, migratory wild birds, international dissemination, clade, South Korea

## Abstract

Highly pathogenic avian influenza H5N6 and H5N1 viruses of clade 2.3.4.4b were simultaneously introduced into South Korea at the end of 2023. An outbreak at a broiler duck farm consisted of concurrent infection by both viruses. Sharing genetic information and international surveillance of such viruses in wild birds and poultry is critical.

Since clade 2.3.4.4 H5Nx highly pathogenic avian influenza (HPAI) viruses first emerged in East Asia in 2013‒14, clade 2.3.4.4b has spread throughout Europe, Africa, and Middle East in 2016‒17, causing >2,000 outbreaks in poultry and wild birds in >30 countries (*1*,*2*). Clade 2.3.4.4b H5N1 viruses were detected in Europe in late 2020; that clade became the predominant subtype in Europe in 2021 and spread throughout Asia and North America. By the end of 2023, H5N1 viruses of that clade had affected bird populations in most of the United States and spread to South America and Antarctica (*3*).

Since early 2014, South Korea has experienced epidemic outbreaks of different subtypes of this clade, including H5N8 in early 2014, 2016‒17, and 2020‒21; H5N6 in 2017‒18; and H5N1 in 2021‒22 and 2022‒23 (*4*–*7*). All those outbreaks in poultry have occurred during the winter season, when migratory birds enter and stay on the Korean peninsula (*8*–*10*). No HPAI virus was detected during regular active surveillance of both wild birds and poultry during May–October 2023.

The first suspected case of HPAI in poultry in the 2023‒24 winter season was reported in South Korea. Surprisingly, birds at that farm were found to be concurrently infected with H5N6 and H5N1 viruses of clade 2.3.4.4b. Subsequently, birds at poultry farms as well as wild birds were found to be infected with H5N6 or H5N1 viruses. Our study analyzed whole-genome sequences of the virus populations of pooled swab samples from the flocks at the farm that were infected with both H5N6 and H5N1 influenza viruses; we defined the farm as the index case. We compared those sequences with the sequences of viruses isolated from other affected farms and wild birds to determine the origins of the viruses and their relationships.

## The Study

**Figure 1:**
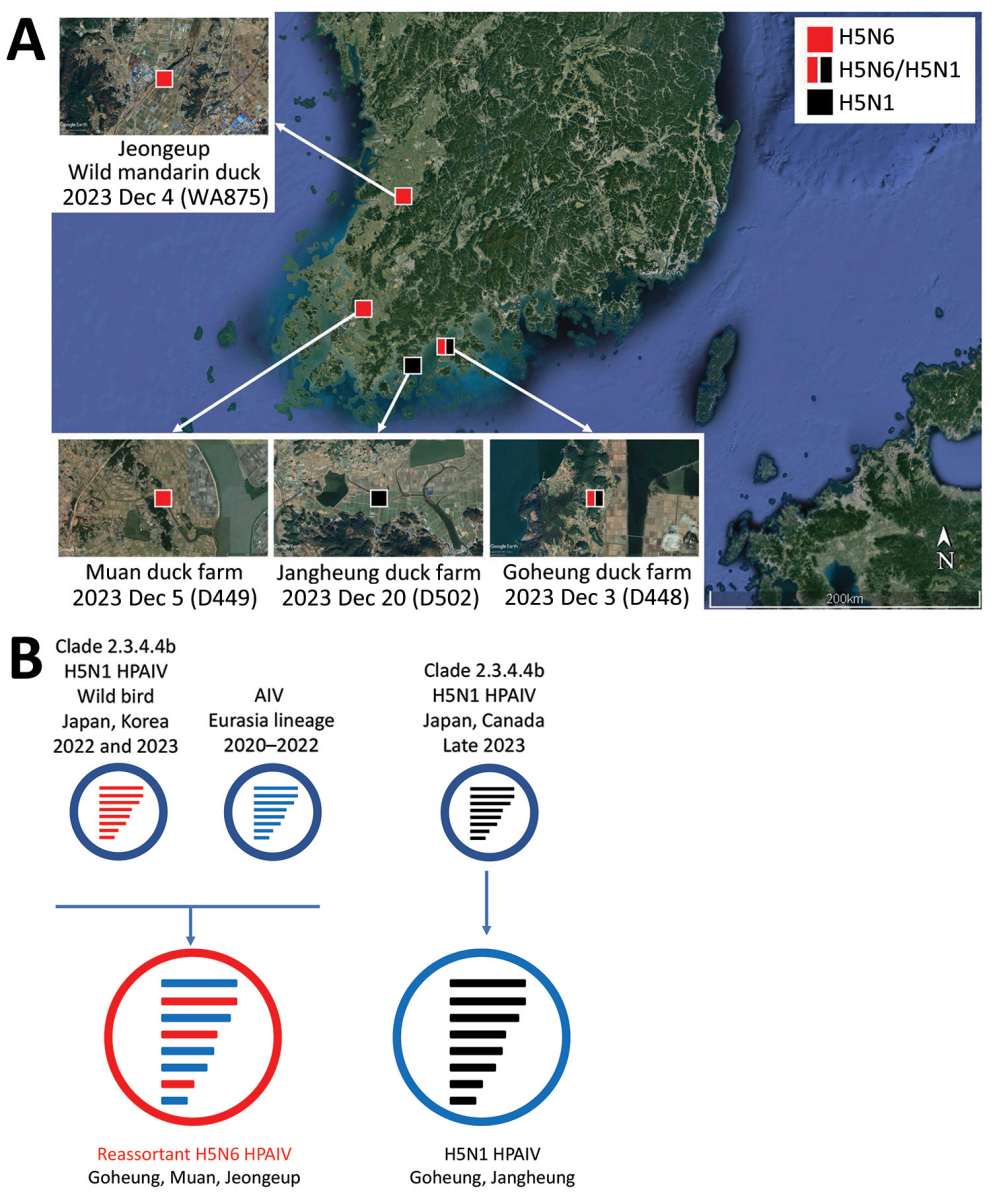
Locations of duck farms and of wild ducks infected with clade 2.3.4.4b HPAIV H5, South Korea, 2023, and their viral genotypes. A) Each square indicates the location of the infected farms or wild birds where the samples were collected. Red indicates H5N6 virus and black H5N1 virus. Satellite image is from Google Earth (https://earth.google.com). B) Genetic constellation of H5N6 and H5N1 viruses concurrently detected in December 2023. The bars represent 8 gene segments of the avian influenza virus in the following order (from top to bottom): polymerase basic 2, polymerase basic 1, polymerase acidic, hemagglutinin, nucleoprotein, neuraminidase, matrix, and nonstructural. The 8 genes of each virus are represented by colored bars, indicating presumed recent donors. HPAIV, highly pathogenic avian influenza virus.

On December 3, 2023, a suspected HPAI infection that caused white diarrhea, reduced feed intake, and increased deaths was reported in 39-day-old broiler ducks at a broiler duck farm (D448) in Goheung, South Korea (Figure 1). We detected matrix and H5 genes in the clinical samples from this index farm by real-time reverse transcription PCR. We determined the deduced amino acid sequence of the HA cleavage site of the H5 genes to be PLREKRRKR/GLF, which indicated high pathogenicity. For the NA gene, we detected both N1 and N6 genes in some flocks at the farm, at which the flocks were separated in different houses. We analyzed co-infection status at that farm by using whole-genome sequences of avian influenza viruses obtained from pooled oropharyngeal swab samples of 20 live ducks from each of 11 flocks using the Nanopore (Oxford Nanopore, https://nanoporetech.com) amplicon sequencing method (Appendix). 

**Figure 2:**
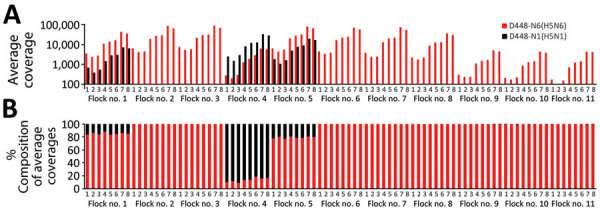
Co-infection status of birds at a broiler duck farm (D448) infected with clade 2.3.4.4b highly pathogenic avian influenza H5N6 and H5N1 viruses in South Korea, December 2023. Reference gene segments for mapping were designated as numbers 1–8; segment 1, polymerase basic 2; segment 2, polymerase basic 1; segment 3, polymerase acidic; segment 4, hemagglutinin; segment 5, nucleoprotein; segment 6, neuraminidase; segment 7, matrix; segment 8, nonstructural. A) Distribution of average coverages of the reads from each pooled oropharyngeal swab sample in the index farm, which had 11 flocks, mapped to the relevant reference viral genomes. Red bars indicate average mapping coverages of A/duck/Korea/D448-N6/2023(H5N6) to its reference gene segments and black bars indicate average mapping coverages of A/duck/Korea/D448-N1/2023(H5N1) to its reference gene segments. Three co-infected flocks (flocks 1, 4, and 5) had sufficient sequencing depth with >1,000-fold coverage of segments 4 (hemagglutinin) and 6 (neuraminidase). B) Distribution of percentage compositions of average coverages transformed from the average coverage values shown in panel A. Birds in flock 4 had more viral reads of H5N1 virus, whereas birds in flocks 1 and 5 had more reads of H5N6 virus. The other 8 flocks had the reads only mapped to H5N6 virus.

We found that 3 flocks, numbers 1, 4, and 5, were co-infected with H5N6 and H5N1 viruses, whereas the other 8 flocks were infected with H5N6 virus only (Figure 2). Analysis of the average coverage at each gene segment as percentage composition showed that birds in flock 4 had more viral reads of H5N1, whereas flocks 1 and 5 had more reads of H5N6 (Figure 2, panel B). We observed the same co-infection pattern in pooled cloacal swabs of flock 4 (data not shown). Because all the swabs from flocks were pooled at sample collection, no clear evidence was found supporting infection with the 2 viruses in a single bird. Because this farm was located very close to the south sea and seawall lake and had a relatively low level of biosecurity, we considered this farm susceptible to virus introduction by migratory birds (Figure 1, panel A).

We detected HPAI H5N6 virus (WA875) in Jeolla-do province in an apparently healthy wild mandarin duck, which we captured and sampled on December 4, 2023, for the active wild bird surveillance program. Two additional broiler-duck farms in the same province were found to be infected with H5N6 (D449) and H5N1 (D502) virus on December 5 and December 20, 2023 (Figure 1, panel A). We assessed the genetic relationships among the HPAI viruses by determining and comparing the complete genome sequences of A/duck/Korea/D448-N6/2023(H5N6), A/duck/Korea/D448-N1/2023(H5N1), A/duck/Korea/D449/2023(H5N6), A/mandarin duck/Korea/WA875/2023(H5N6), and A/duck/Korea/D502/2023(H5N1). Their sequences have been deposited in GISAID (https://www.gisaid.org; accession nos. EPI_ISL_18819959–61, EPI_ISL_18819826, and EPI_ISL_18819797).

The H5N6 viruses, D448-N6, D449, and WA875, showed high nucleotide sequence identities in all 8 genes among them (>99.8%). The sequences of their polymerase basic (PB) 1, hemagglutinin (HA), and matrix (M) genes were very close (99.53%–99.83%) to the respective genes of clade 2.3.4.4b HPAI H5N1 viruses isolated from wild birds in Japan and South Korea in 2022‒23. The 4 internal genes of the H5N6 viruses, PB2, polymerase acidic protein (PA), nucleoprotein (NP), and nonstructural protein (NS), were closely related to the respective genes found in the Eurasian low pathogenicity avian influenza (LPAI) viruses of diverse subtypes isolated from wild birds in 2020 and 2022. Their N6 genes appeared to be close to the poultry viruses isolated in East Asia in 2021 and 2022, although the nucleotide identities were relatively low (98.1%–98.62%) (Table 1). Of interest, the protein encoded by the N6 gene in the isolates from this study had a deletion of 12 aa residues at positions 58–69; this neuraminidase (NA) stalk deletion has been often observed in poultry-adapted viruses (*11*,*12*). From the avian influenza active surveillance program in South Korea during 2019–2023, N6 genes were detected only in LPAI viruses isolated from wild birds; we did not observe this NA stalk deletion among the analyzed viruses (data not shown).

**Table 1:** Nucleotide sequence identities of gene segments between reassortant clade 2.3.4.4b HPAI H5N6 virus from a duck farm in South Korea, 2023, compared with A/duck/Korea/D448-N6/2023(H5N6) virus and the closest referent in the GISAID database*

Gene	Virus	GISAID accession no.	% Identity	Sample date	LPIAI or HPAI H5 clade
PB2	A/mallard/South_Korea/20X-20/2021(H7N9)	EPI_ISL_6781375	98.73	2021 Jan 6	LPAI
PB1	A/large billed_crow/Kanagawa/1403C006/2023(H5N1)	EPI_ISL_17949961	99.69	2023 Mar 10	2.3.4.4b
PA	A/common_teal/Amur_region/92b/2020(H6N2)	EPI_ISL_1184535	99.58	2020 Sep 6	LPAI
HA	A/Mandarin_duck/Korea/WA496/2022(H5N1)	EPI_ISL_15647836	99.53	2022 Oct 10	2.3.4.4b
NP	A/gadwall/Novosibirsk_region/3407k/2020(H4N6)	EPI_ISL_1184520	98.8	2020 Aug 29	LPAI
NA	A/duck/Hunan/S40199/2021(H5N6)†	EPI_ISL_11208196	98.62	2021 Dec 1	2.3.4.4b
M	A/Mandarin_duck/Korea/WA496/2022(H5N1)	EPI_ISL_15647836	99.8	2022 Oct 10	2.3.4.4b
NS	A/Falcated_duck/South_Korea/JB42–30/2020(H9N2)	EPI_ISL_4072076	99.64	2020 Feb 19	LPAI

*One A/duck/Korea/D448-N6/2023(H5N6) isolate from a wild bird (WA875) and 2 isolates from poultry (D448-N6, D449) had high nucleotide identities among them in all 8 genes: PB2, >99.82%; PB1, >99.78%; PA, >99.86%; HA, >99.82%; NP, >99.87%; NA, >99.86%; M, >99.90%; NS, >99.88%. GISAID, https://www.gisaid.org; HA, hemagglutinin; HPAI, highly pathogenic avian influenza; LPAI, low pathogenicity avian influenza; M, matrix; N, nucleocapsid; NA, neuraminidase; NP, nucleoprotein; NS, nonstructural; PA, polymerase acidic; PB, polymerase basic. †This virus was isolated from bird in poultry market, and all the other viruses compared were from wild birds.

We found no HPAI H5N6 viruses showing nucleotide similarities >98.5% in all of the 8 genes at once to the new H5N6 isolates in the public databases. However, a wild bird isolate from Japan (A/peregrine falcon/Saga/4112A002/2023, EPI_ISL_18740267) that was collected on December 6, 2023, was almost identical to the H5N6 Korean viruses (T. Hiono, pers. comm., email, 2024 Jan 11), suggesting that these emerged viruses spread coincidently throughout this winter in East Asia.

The nucleotide sequences of the coding regions of 2 poultry H5N1 viruses, D448-N1 and D502, were very similar (>99.0%) and were very closely related to the sequences of clade 2.3.4.4b H5N1 viruses circulating in Japan and Canada in 2023 (Table 2; Appendix Figures 1–5,7–9). Those clade 2.3.4.4b HPAI H5N1 viruses of diverse genotypes have been prevalent in Europe and North America (*3*) and had been introduced into South Korea during the epidemics of 2021–22 and 2022–23 (*5*,*8*). We did not detect significant mutations related to mammal adaptation or antiviral resistance in the newly isolated H5N6 and H5N1 HPAI viruses.

**Table 2:** Nucleotide sequence identities of gene segments between the clade 2.3.4.4b HPAI H5N1 virus from a duck farm in South Korea, 2023, compared with A/duck/Korea/D448-N1/2023(H5N1) virus and the closest referent in the GISAID database*

Gene	Virus†	GISAID accession no.	% Identity	Sample date	H5 clade
PB2	A/large-billed_crow/Hokkaido/B067/2023(H5N1)	EPI_ISL_18591747	99.78	2023 Sep 23	2.3.4.4b
PB1	A/Eurasian_wigeon/Kagoshima/4611J002/2023(H5N1)	EPI_ISL_18603583	99.87	2023 Sep 12	2.3.4.4b
PA	A/goose/Magadan/2272–5/2022(H5N1)	EPI_ISL_18071580	99.81	2022 Oct 09	2.3.4.4b
HA	A/Eurasian_wigeon/Kagoshima/4611J002/2023(H5N1)	EPI_ISL_18603583	99.53	2023 Sep 12	2.3.4.4b
NP	A/large-billed_crow/Hokkaido/0103E088/2023(H5N1)	EPI_ISL_17950087	99.87	2023 Mar 30	2.3.4.4b
NA	A/canada_goose/BC/AIVPHL-371/2023(H5N1)	EPI_ISL_17051482	99.79	2023 Jan 16	2.3.4.4b
M	A/northern_pintail/Okayama/331A003/2023(H5N1)	EPI_ISL_18603584	99.9	2023 Sep 13	2.3.4.4b
NS	A/Barnacle_Goose/Netherlands/8/2022(H5N1)	EPI_ISL_10347219	99.76	2022 Jan 28	2.3.4.4b

*Two A/duck/Korea/D448-N1/2023(H5N1) isolates from poultry (D448-N1, D502) had high nucleotide identities between them in all 8 genes, PB2, 99.52%; PB1, 99.78%; PA, 99.72%; HA, 99.30%; NP, 99.73%; NA, 99.22%; M, 99.80%; NS, 99.05%. GISAID, https://www.gisaid.org; HA, hemagglutinin; HPAI, highly pathogenic avian influenza; M, matrix protein; N, nucleocapsid protein; NA, neuraminidase; NP, nucleoprotein; NS, nonstructural protein; PA, polymerase acidic protein; PB, polymerase basic protein. †All the viruses compared were isolated from wild birds.

## Conclusions

This study describes the simultaneous introduction of H5N1 virus and a new reassortant H5N6 HPAI virus of clade 2.3.4.4b into South Korea in 2023. Better understanding of this spatial and genomic dynamic requires enhanced and timely sharing of genetic information and international surveillance of HPAI and LPAI viruses in wild birds and poultry.

Appendix Additional information about concurrent infection with clade 2.3.4.4b H5N6 and H5N1 highly pathogenic avian influenza viruses, South Korea, 2023.
