# Supplementary material for: Concurrent Infection with Clade 2.3.4.4b Highly Pathogenic Avian Influenza H5N6 and H5N1 Viruses, South Korea, 2023
Source: Emerg Infect Dis. 2024 Jun;30(6):1223–7. doi: 10.3201/eid3006.240194 (PMC11138991; doi:10.3201/eid3006.240194)
Supplement: Appendix — Additional information about concurrent infection with clade 2.3.4.4b H5N6 and H5N1 highly pathogenic avian influenza viruses, South Korea, 2023. [file 24-0194-Techapp-s1.pdf]

Article DOI: http://doi.org/10.3201/eid3006.240194

EID cannot ensure accessibility for supplementary materials supplied by authors. Readers who have difficulty accessing supplementary content should contact the authors for assistance.

Concurrent Infection with Clade 2.3.4.4b Highly Pathogenic Avian Influenza H5N6 and H5N1 Viruses, South Korea, 2023

Appendix

Methods

Surveillance and Sample Collection

On 3 December 2023, a broiler duck farm owner in Goheung, Jeolla-do, reported an increase in mortality of 39 day-old broiler ducks, to the municipal administration. Staff members of the veterinary service in Jeolla-do visited this farm and collected oropharyngeal and cloacal swab samples from 20 birds in each flock. The swabs were pooled by flock in sample collection phase. All of these pooled swabs were H5 positive, and the samples were transferred to the Animal and Plant Quarantine Agency (APQA) for confirmatory diagnosis. For AI active surveillance program on wild bird, on 4 December 2023, staff members at the Livestock Health Control Association, the organization supporting government for sampling, disease control and sanitation, captured 29 live wild mandarin ducks in Jeongeup in Jeolla-do by mist net and collected oropharyngeal and cloacal swab samples from each, then released the wild birds at the site of capture. The samples were delivered to the APQA the next day, with H5N6 HPAI viruses subsequently isolated from oropharyngeal swabs of two mandarin ducks (WA875 and WA859). Active periodic virological surveillance by the veterinary service of Jeolla-do on 5 and 20 December, 2023, found that birds at two broiler duck farms in Muan and Jangheung, respectively, were infected with H5 viruses. Following transfer to the APQA, these positive samples were confirmed to be H5N6 and H5N1 HPAIs, respectively.

Subtyping and Pathotyping

All the samples delivered to APQA were analyzed by qRT-PCR using VDx® AIV qRT-PCR Ver 2.1 (NP-AIV-37, NP-AIV-38, NP-AIV-39, Median Diagnostics, Gangwon, Korea), targeting the AIV matrix and the H5 and H7 genes according to the manufacturer's protocol. H5 positive samples were subjected to in-house HA/NA subtyping by endpoint RT-PCR. Briefly, nucleic acids were extracted from the samples using NX-48 Viral NA kits with Nextractor ® NX-48 (Genolution, Seoul, Korea). Amplicons of eight gene segments of the viruses in these samples and virus isolates were generated by one-step RT-PCR using a previously described protocol (*1*). Gene sequences were amplified using a SuperScript III One-Step RT-PCR System with Platinum Taq DNA Polymerase (12574–026, Invitrogen, Carlsbad, CA, USA), two forward primers; CommonA-Uni12G (5′-GCCGGAGCTCTGCAGATATCAGCGAAAGCAGG-3′) and CommonA-Uni12 (5′-GCCAGAGCTCTGCAGATATCAGCAAAAGCAGG-3′), and one reverse primer; CommonA-Uni13G (5′-

GCCGGAGCTCTGCAGATATCAGTAGAAACAAGG-3′). Each reaction mixture contained 25 μL 2X RT-PCR buffer, 2 μL commercial reverse transcription and PCR polymerase, 0.5 μL of each forward primer (10 μM), 1 μL of reverse primer (10 μM), 10 μL of RNA extracted from a target sample, and 11 μL nuclease free water. The amplification conditions consisted of heating at 42°C for 15 minutes, 55°C for 15 minutes, 60°C for 5 minutes, and 94°C for 2 minutes (ramp rate: 2.5°C/s); 5 cycles of denaturation at 94°C for 30 seconds, annealing at 45°C for 30 seconds (ramp rate: 2.5°C/s) and extension at 68°C for 5 minutes (ramp rate: 0.5°C/s); 37 cycles of denaturation at 94°C for 30 seconds, annealing at 55°C for 30 seconds and extension 68°C for 5 minutes; and a final extension at 68°C for 5 minutes (ramp rate: 2.5°C/s). For PCR purification, Each RT-PCR product was mixed with 90 μL of Ampure XP SPRI Reagent (A63882, Beckman Coulter, La Brea, CA, USA) and quantified using Qubit dsDNA BR assay kits (Q32853, Invitrogen) with a Qubit 4 fluorometer (Q33226, Invitrogen). Sequencing libraries were generated from each quantified RT-PCR product using a Native Barcoding Kit 96 V14 (SQK-NBD114.96, Oxford Nanopore Technology, Oxford, UK), according to the manufacturer's instructions. All libraries were sequenced on a MinION Mk1C (MIN-101C, Oxford Nanopore Technology) using a Flow Cell (R10.4.1) (FLO-MIN114, Oxford Nanopore Technology) according to the manufacturer's instructions.

Generation of Consensus Genome Sequences

Raw reads were collected for basecalling using MinKNOW V. 23.11.3, with the options, 'Enable read splitting: on', 'Override read splitting min score: on', 'Minimum read splitting score: 58', 'Flow cell product code: FLO-MIN114', 'Chemistry: DNA-400bps-5kHz', 'Model: Super-accurate basecalling', 'Modified basecalling: None', 'Barcoding kits: SQK-NBD114–96', 'Trim barcodes: on', 'Barcode both ends: on', 'Mid-read barcode filtering: off', 'Override minimum barcoding score: off', and 'Alignment reference: off'. Only passed reads were retained for further analysis.

Sequences were assembled using CLC Genomics workbench 23.0.4. Initial import settings followed the options 'Paired reads: off', 'Discard read names: on', 'Discard quality score: off', and 'Illumina options: off'. Reads were mapped to the reference sequences using the default parameters of CLC Genomics workbench 23.0.4, including 'Match score: 1', 'Mismatch cost: 2', 'Cost of insertions and deletions: Linear gap cost', 'Insertion cost: 3′, 'Deletion cost: 3′, 'Insertion open cost: 6', 'Insertion extend cost: 1', 'Deletion open cost: 6', 'Deletion extend cost: 1', 'Length fraction: 0.5′, 'Similarity fraction: 0.8', 'Global alignment: off', 'Auto-detect paired distances: on', 'Non-specific match handling: Map randomly' and 'Masking mode: No masking'. Local realignment was performed using the options 'Realign unaligned ends: on', 'Multi-pass realignment: 2', and 'Guidance-variant settings: off'. Consensus sequences were extracted using the options 'Low coverage definition threshold: 0', 'Low coverage handling: Remove regions with low coverage and Join after removal', and 'Conflict resolution: Vote'. All the sequence data determined in this study were registered in Global Initiative on Sharing Avian Influenza Data (GISAID) under the sequences EPI_ISL_18819959 for A/duck/Korea/D448-N6/2023(H5N6), EPI_ISL_18819960 for A/duck/Korea/D448-N1/2023(H5N1), EPI_ISL_18819961 for A/duck/Korea/D449/2023 (H5N6), EPI_ISL_ 18819826 for A/mandarin

duck/Korea/WA875/2023(H5N6), and EPI_ISL_18819797 for A/duck/Korea/D502/2023(H5N1).

Pairwise Comparisons

Nucleotide identity between viral gene segments of A/duck/Korea/D448-N6/2023(H5N6) and A/duck/Korea/D448-N1/2023(H5N1) were determined by comparing coding region sequence. The percent nucleotide identifies for segments 1–5 and 7–8 were found to be 91.1%, 94.8%, 95.5%, 97.9%, 92.6%, 99.2%, and 95.7%, respectively. Segment 6 in the two viruses were found to be of different subtypes.

Phylogenomic Analysis

Reference nucleotide sequences in the GISAID databases, specifically the isolates in the 100 top BLAST hits for the eight genes of the isolated HPAI viruses, were downloaded by BLAST searches on 30 December 2023. These sequences were aligned with MAFFT using the default parameters for FASTA alignment. All untranslated regions (UTRs) were removed, with only protein-coding sequences of each segment being retained. Phylogenetic analyses of the HA gene and the N1 and N6 subtypes of the NA gene were performed using 518, 362, and 141 sequences, respectively; whereas phylogenetic analyses of the PB2, PB1, PA, NP, MP, and NS genes were performed using 802, 795, 797, 809, 804, and 775 sequences, respectively. Maximum likelihood trees based on the aligned sequences were constructed using RAxML on XSEDE version 8.2.12 (*2*) and CIPRES Science Gateway (https://www.phylo.org/) (*3*) Trees were displayed with interactive iTOL (https://itol.embl.de/login.cgi), with bootstrap values over than 75% noted on the nodes (*4*)

References

1. Van Poelvoorde LAE, Bogaerts B, Fu Q, De Keersmaecker SCJ, Thomas I, Van Goethem N, et al. Whole-genome-based phylogenomic analysis of the Belgian 2016–2017 influenza A(H3N2) outbreak season allows improved surveillance. Microb Genom. 2021;7:000643. PubMed https://doi.org/10.1099/mgen.0.000643

2. Stamatakis A. RAxML version 8: a tool for phylogenetic analysis and post-analysis of large phylogenies. Bioinformatics. 2014;30:1312–3. PubMed https://doi.org/10.1093/bioinformatics/btu033

3. Miller MA, Schwartz T, Pickett BE, He S, Klem EB, Scheuermann RH, et al. A RESTful API for access to phylogenetic tools via the CIPRES science gateway. Evol Bioinform Online. 2015;11:43–8. PubMed https://doi.org/10.4137/EBO.S21501

4. Letunic I, Bork P. Interactive Tree Of Life (iTOL) v5: an online tool for phylogenetic tree display and annotation. Nucleic Acids Res. 2021;49(W1):W293–6. PubMed https://doi.org/10.1093/nar/gkab301

Appendix Figures (following pages). Sequences of H5Nx viruses. Red and black squares indicate H5N6 and H5N1 viruses isolated in Korea in December 2023. Black circles indicate H5Nx viruses isolated in Korea from 2020 to July 2023. Sequences determined in this study are shown in bold.

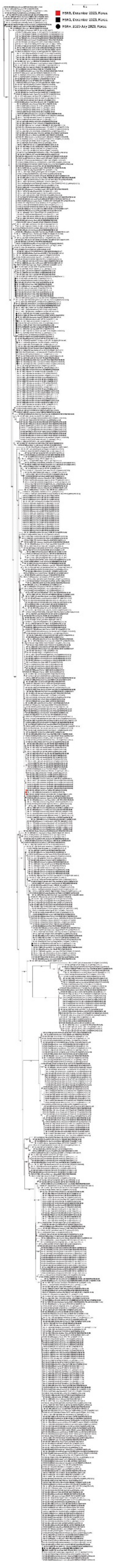

Appendix Figure 1. Phylogenetic tree of PB2 gene.

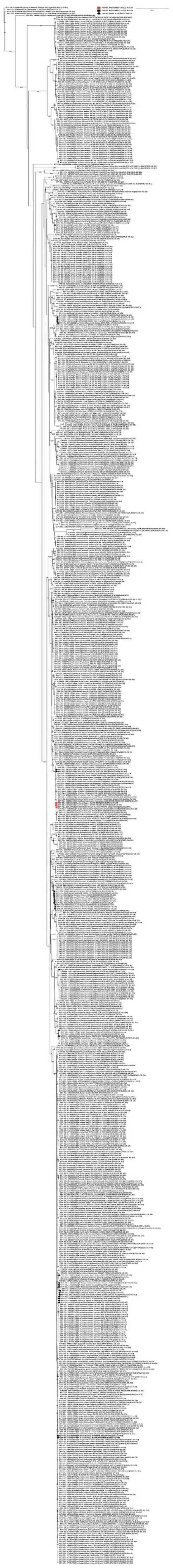

Appendix Figure 2. Phylogenetic tree of PB1 gene.

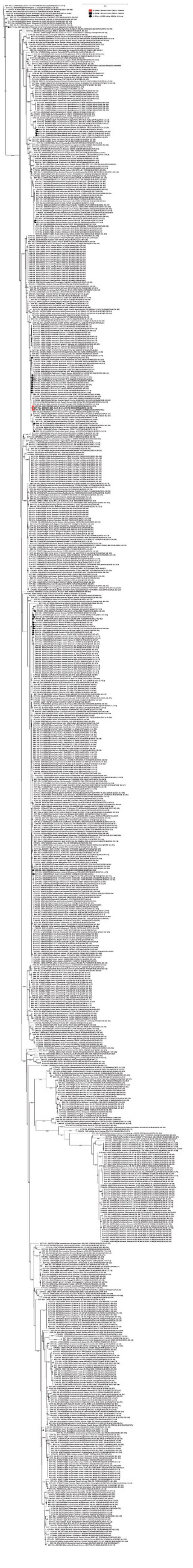

Appendix Figure 3. Phylogenetic tree of PA gene.

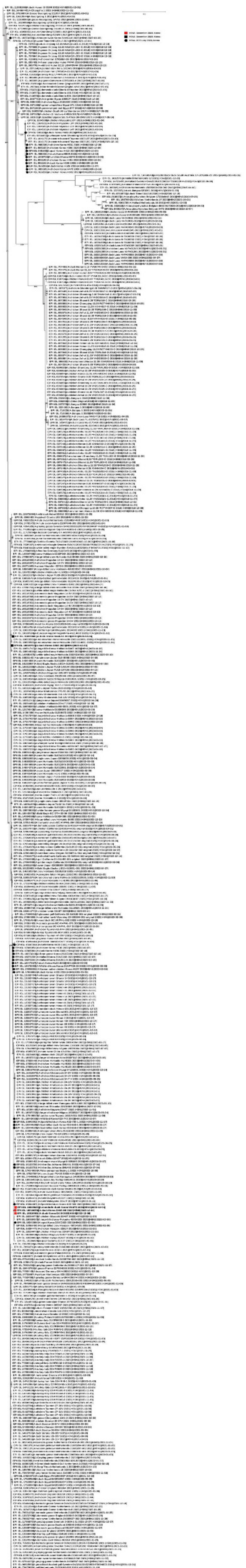

Appendix Figure 4. Phylogenetic tree of HA gene.

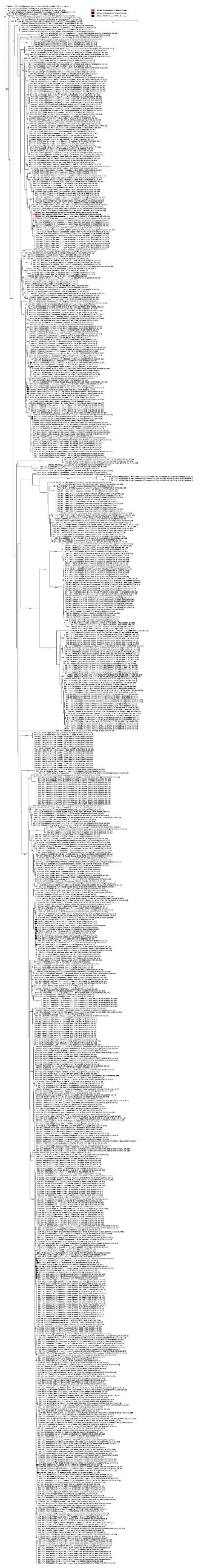

Appendix Figure 5. Phylogenetic tree of NP gene.

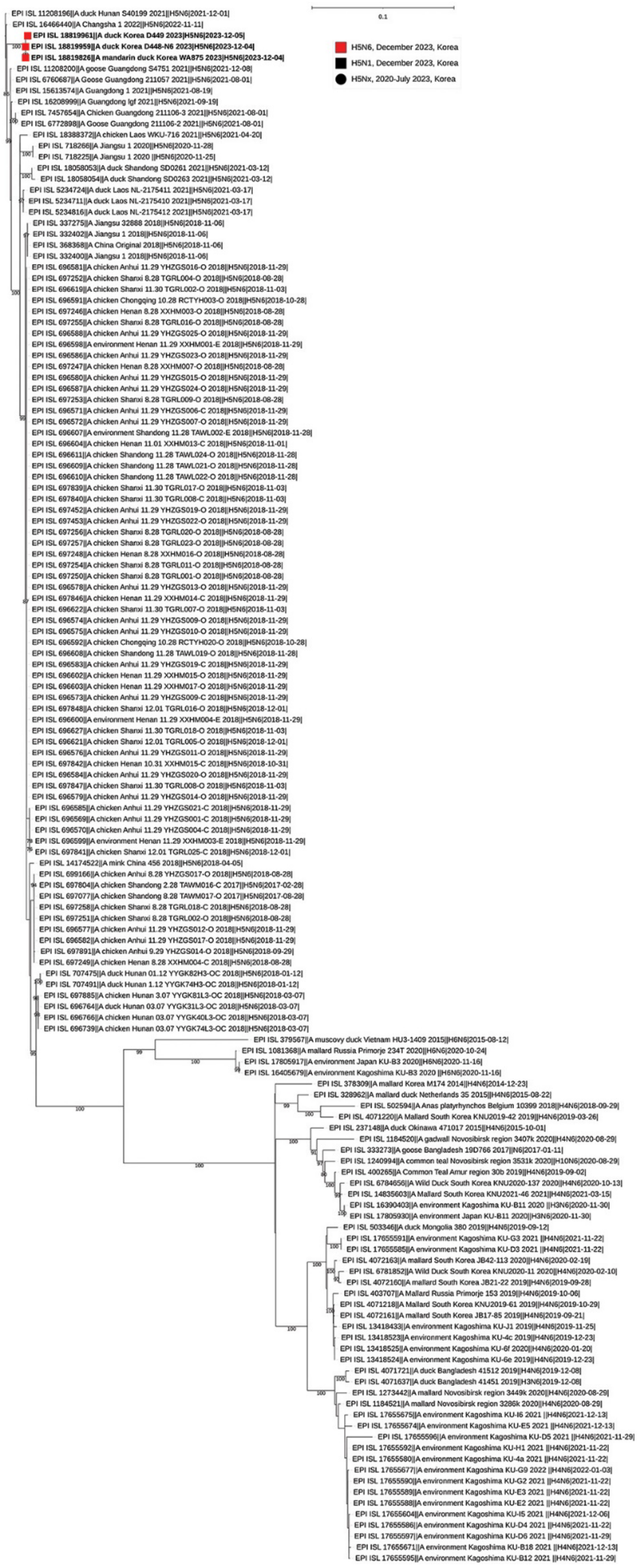

Appendix Figure 6. Phylogenetic tree of NA gene of N6 subtype.

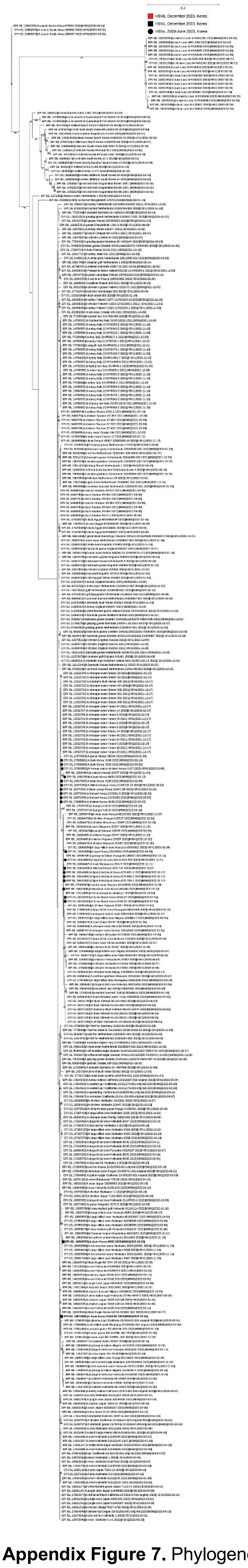

Appendix Figure 7. Phylogenetic tree of NA gene of N1 subtype.

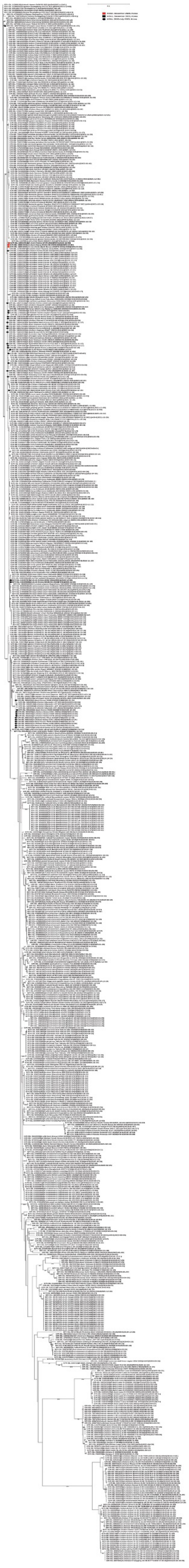

Appendix Figure 8. Phylogenetic tree of MP gene.

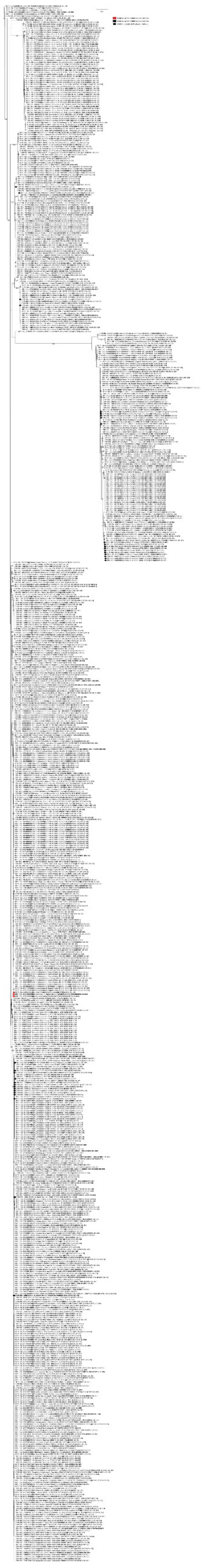

Appendix Figure 9. Phylogenetic tree of NS gene.
